# Biodegradation of Typical Plastics: From Microbial Diversity to Metabolic Mechanisms

**DOI:** 10.3390/ijms25010593

**Published:** 2024-01-02

**Authors:** Shiwei Lv, Yufei Li, Sufang Zhao, Zongze Shao

**Affiliations:** 1 Key Laboratory of Marine Genetic Resources, Third Institute of Oceanography, Ministry of Natural Resources of China, Xiamen 361005, China; 22b329013@stu.hit.edu.cn (S.L.); 2111210022@email.cugb.edu.cn (Y.L.); 23b330004@stu.hit.edu.cn (S.Z.); 2 School of Environmental Science, Harbin Institute of Technology, Harbin 150090, China; 3 School of Marine Sciences, China University of Geosciences, Beijing 100083, China; 4 Southern Marine Science and Engineering Guangdong Laboratory (Zhuhai), Zhuhai 519000, China

**Keywords:** plastic biodegradation, marine ecosystem, microbial diversity, degradation pathways, key enzymes

## Abstract

Plastic production has increased dramatically, leading to accumulated plastic waste in the ocean. Marine plastics can be broken down into microplastics (<5 mm) by sunlight, machinery, and pressure. The accumulation of microplastics in organisms and the release of plastic additives can adversely affect the health of marine organisms. Biodegradation is one way to address plastic pollution in an environmentally friendly manner. Marine microorganisms can be more adapted to fluctuating environmental conditions such as salinity, temperature, pH, and pressure compared with terrestrial microorganisms, providing new opportunities to address plastic pollution. Pseudomonadota (Proteobacteria), Bacteroidota (Bacteroidetes), Bacillota (Firmicutes), and Cyanobacteria were frequently found on plastic biofilms and may degrade plastics. Currently, diverse plastic-degrading bacteria are being isolated from marine environments such as offshore and deep oceanic waters, especially *Pseudomonas* spp. *Bacillus* spp. *Alcanivoras* spp. and Actinomycetes. Some marine fungi and algae have also been revealed as plastic degraders. In this review, we focused on the advances in plastic biodegradation by marine microorganisms and their enzymes (esterase, cutinase, laccase, etc.) involved in the process of biodegradation of polyethylene terephthalate (PET), polystyrene (PS), polyethylene (PE), polyvinyl chloride (PVC), and polypropylene (PP) and highlighted the need to study plastic biodegradation in the deep sea.

## 1. Introduction

Plastic is an organic synthetic polymer material that is used worldwide. It has gradually replaced traditional materials such as wood, metal, and glass in a variety of applications due to its low cost, durability and high strength compared to traditional materials [1,2,3]. Based on polymer demand in Europe, six common plastic polymers are polyethylene (PE), 29.8%; polypropylene (PP), 19.3%; polyvinyl chloride (PVC), 10.2%; polyurethane (PUR), 7.7%; polyethylene terephthalate (PET), 7.4%; and polystyrene (PS), 6.6% [4]. Currently, plastic production is approximately 450 million metric tons per year globally [5]. Most plastics are poorly managed and turn into plastic waste that enters the environment. The global plastic waste production in 2022 was approximately 380 million tons [6]. Of this, approximately 8 million tons of plastic waste was released into the ocean [7]. Therefore, the accumulation of plastic waste in the ocean continues to increase [8].

Plastic products are usually highly stable for long-term use and can last for decades in the environment [9], and they can be broken down into microplastics or even nanoplastics using different processes (UV, thermal degradation, mechanical action and human activities, etc.) [10,11]. Microplastics in the ocean pose serious threats to marine ecosystems. The accumulation of microplastics has been found in many marine organisms, such as fish [12,13], oysters [14], and scallops [15], crossing various biological barriers to reach organs and disrupting cellular metabolic activity. Nanomicroplastics are usually more elusive, with higher threats leading to cellular oxidative stress and metabolic disorder [16,17]. These newly emerging pollutants are troublesome to marine ecological health. For example, microplastics can hinder cyanobacterial growth by inhibiting photosynthesis [18,19] and causing cell damage [20].

In addition, a variety of chemical additives that are added to plastics to alter their properties, including plasticisers, flame retardants, ultraviolet stabilisers, and antioxidants, also cause environmental threats [21,22]. Additives are poorly chemically bound and released into seawater as the material degrades and weathers [23]. There are many types of plasticisers, and phthalic acid esters (PAEs) are widely consumed, accounting for approximately 70% of the global plasticiser market [24,25]. In particular, di(2-ethylhexyl) phthalate (DEHP) is often used as a plasticiser for PVC, accounting for 51% of global phthalate consumption [26]. PAEs are classified as priority pollutants by the United States Environmental Protection Agency (USEPA) due to their acute toxicity [27]; they are toxic to aquatic animals and, more importantly, carcinogenic to humans [28]. Research on flame retardants has focused on brominated flame retardants (BFRs) and organophosphorous flame retardants (OPFRs). Brominated flame retardants have become the largest market group due to their high performance and low cost [24], while organophosphorus flame retardants are also growing at a rate of 4.6% per year [29]. It has been shown that flame retardants can cause decreased immunity, metabolic disorders, and endocrine disruption in marine organisms [30,31]. Bisphenol A (BPA) is commonly used as a stabiliser and antioxidant for plastic and as a monomer for polycarbonate (PC) [32]. As a result, BPA is a widely used chemical that is often detected in water. BPA can cause severe endocrine disruption in marine organisms [33] and impair reproduction under prolonged exposure [34]. Therefore, both microplastics and plastic additives impose threats to animals, plants, and microalgae in marine ecosystems.

Several approaches have been applied for plastic waste treatment including incineration, chemical recycling, landfilling, and mechanical reprocessing [8]. As shown in Table 1, incineration of plastic garbage has serious drawbacks, as toxic combustion products (e.g., hazardous dioxins) can be released and cause air pollution. The major problem of chemical recycling however is its cost, and also the production of toxic products [35]. Landfill can cause some environmental impact, such as releasing odorous components and greenhouse gases or polluting the land and aquifer by releasing leachable components [36]. Mechanical reprocessing requires pre-sorting and damages their mechanical performances [37]. Therefore, an effective and environment-friendly method to deal with the accumulation of plastic waste is required.

**Figure 1:**
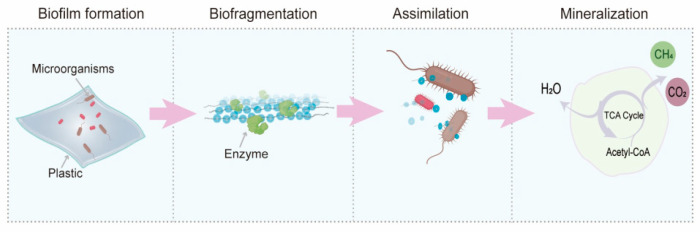
Microbial degradation pathways of plastic debris.

Microbial depolymerisation and biodegradation is an alternative way to reduce plastic waste in the environment by recycling and treating environmental waste in the future. Plastic microbial degradation involves steps of biodeterioration, biofragmentation, assimilation, and mineralisation [39] (Figure 1). In the first phase, microorganisms with plastic degrading abilities attach to the plastic surface to form biofilms. In the second phase, extracellular enzymes are secreted from the microbial biofilms, which convert long-chain polymers into oligomers and oxidise them under the influence of oxygen to form carbonyl, carboxyl, hydroxyl, and other oxygen-containing functional groups [40]. Next, oligomers are assimilated through in vivo metabolism mechanisms (mostly β-oxidation mechanisms and the citric acid cycle) by microorganisms. Finally, the oligomers are mineralised to form CO_2_, CH_4_, and H_2_O [5,41]. Compared with other degradation processes, biodegradation is a cost-effective and eco-friendly method. In this review, we focused on the biodegradation of synthetic plastics by marine bacteria, fungi, and algae and the enzymes involved in this process, which are important in attenuating plastic pollution in situ.

## 2. Bibliometric Study

**Figure 2:**
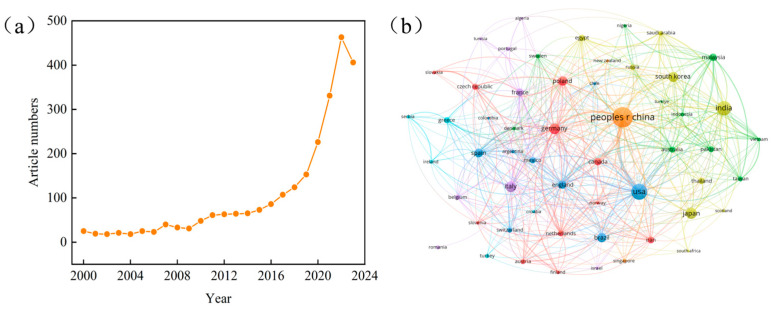
Bibliometric study showing: (**a**) number of articles published during the period of 2000 to 2023; (**b**) map of country cooperation in treatment of plastic pollution from WoS.

To understand the research trends in the present topic, a bibliometric study, based on data obtained from Web of Science (WoS), was carried out. A total of 2648 publications were obtained with the keyword “plastic biodegradation”. The annual production of publications is shown in Figure 2a; the number of articles showed little growth until 2006. The number of articles started to increase annually during 2007–2017. Since 2018, there has been a rapid increase in articles on plastic biodegradation. A total of 1703 articles, accounting for 64.3% of the outputs, were published between January 2018 and December 2023. The increase in the number of published articles indicates that significant progress has been made in plastic biodegradation research in recent years. But the process of bioremediation might have some process hindrances, as factors like time for incubation, process of degradation have not been optimised.

Figure 2b shows the countries collaboration network. China is the most active country in plastic biodegradation research. Chinese researchers have published 526 papers, accounting for 19.86% of the total. The other countries on plastic biodegradation research are the United States, India, Japan, and Germany, accounting for 12.73%, 9.93%, 5.85%, and 5.62%, respectively. Due to rapid economic growth, the plastic consumption in developing countries is higher than the global average. According to statistics, Asia’s global plastics production in 2021 was 390.7 million tons, and China has the highest contribution to plastic consumption [42]. Based on plastic consumption and government support for plastic biodegradation research, the ranking seems reasonable.

## 3. Microbial Degradation of Plastics

### 3.1. Bacteria

Bacteria are noted to be the most important and abundant organisms in nature and can degrade plastics. In the last few years, plastic-degrading bacteria have been isolated from a wide range of habitats, such as marine, landfill, soil, and compost habitats [43].

#### 3.1.1. *Pseudomonas* Species

Currently, among the different bacterial genera associated with plastic degradation, *Pseudomonas* spp. account for 21% [44]. The earliest microbial studies on plastic biodegradation also started with *Pseudomonas* spp. [45]. Different species of *Pseudomonas* spp. have been used for the degradation of plastics. Among them, *Pseudomonas aeruginosa* has received much attention and has been confirmed to degrade various plastics. *P. aeruginosa* isolated from the intestines of superworms showed daily weight loss of 0.64%, 0.098%, and 0.025% for PE, PS, and PP, respectively [46]. *P. aeruginosa* isolated from waste dumps showed polyethylene (PE) degradation rates of 6.5% and 8.7% after 60 days of incubation in minimal salt medium (MSM) and Bushnell–Haas broth (BHM), respectively [47]. *P. aeruginosa* isolated from sewage water-contaminated surface soil incubated in nutrient broth (NB) medium for one month PP showed a weight loss of 5.37% [48]. Moreover, other species of the *Pseudomonas*, such as *Pseudomonas citronellolis* [49], *Pseudomonas putida* [50], *Pseudomonas alcaligenes* [51], and *Pseudomonas fluorescens* [52], have also been shown to degrade different types of plastics.

In the marine environment, *Pseudomonas* spp. have been widely detected on plastic biofilms, but there have been fewer reports of their strains being isolated individually to degrade plastic compared with terrestrial sources [53,54]. *Pseudomonas aestusnigri* isolated from marine sand samples could effectively degrade PET with a novel carboxylic acid ester hydrolase [55,56]. *Pseudomonas rhodesiae* isolated from Brazilian deep-sea sediments could form biofilms on high density polyethylene (HDPE) and cause structural changes in the plastic [57]. Microbial consortia formulated by *P. putida* and *P. stutzeri* isolated from Bangalore Lake could degrade low-density polyethylene (LDPE), which showed higher degradability and 90% weight loss after 40 days of incubation [58].

#### 3.1.2. *Bacillus* Species

*Bacillus* spp. can effectively degrade different types of plastics, represented by *Bacillus cereus*, *Bacillus safensis*, and *Bacillus subtilis*. For example, *B. cereus* isolated from mangroves in Peninsular Malaysia resulted in weight losses of 1.6%, 6.6%, and 7.4% for PE, PET, and PS, respectively, in 40 days [59]. *B. subtilis* H1584 isolated from pelagic waters degraded LDPE, which led to a weight loss of 1.75% in 30 days [60]. *B. subtilis* isolated from seawater 0–30 cm depth showed a weight loss of 1.54% for LDPE after 90 days of incubation [61]. *Bacillus paralicheniformis* G1 isolated from deep-sea sediment was found to degrade PS by approximately 18% in the first 30 days and reached 34% in 60 days [62].

By far, plastic-degrading *Bacillus* isolated from terrestrial environments are more diverse; for example, *B. cereus* isolated from landfills showed a 1.78% degradation rate of HDPE in 30 days [63], and the isolate of *B. cereus* from cattle manure caused a weight loss of 5.9% HDPE in 83 days [64]. *B. safensis* from landfill soil caused 8% weight loss of PLA 1006 in 30 days [65]. Two strains of *B. amyloliquefaciens* (BSM-1) and *B. amyloliquefaciens* (BSM-2) isolated from municipal solid soil showed 11% and 16% degradation rates of LDPE, respectively, after 60 days of incubation [66].

#### 3.1.3. *Alcanivorax* Species

Species within the genus *Alcanivorax* are widely distributed in the ocean and are well-known hydrocarbon degraders [67]. Their recurrence in biofilms of marine plastics also predicted their capacity to degrade plastics [68,69]. *Alcanivora* sp. 24 isolated from marine plastic litter could reduce mass by 0.9% after 34 days of incubation with LDPE [70]. *Alcanivorax borkumensis* isolated from the Mediterranean Sea could form biofilms on LDPE, with a significant weight loss of 3.5% after 80 days [71]. *Alcanivorax wenustensis* isolated from the deep sea could degrade polycaprolactone (PCL) and make its surface rough [72]. *Alcanivorax xenomutans* isolated from mangroves could effectively degrade PS by observing the amount of strain growth [73].

#### 3.1.4. Actinomycetes

Currently, actinomycetes that degrade plastics are mainly isolated from soil [74]. However, some actinomycetes have also been isolated from marine environments that can effectively degrade plastics. *Kocuria palustris* isolated from Arabian Sea seawater could reduce PE weight by 1% after 75 days of incubation [60]. *Rhodococcus ruber* isolated from mangrove sediment degraded PP with 6.4% weight loss after 40 days of incubation [75]. *Rhodococcus pyridinivorans* P23 isolated from deep-sea sediment could reduce the weight of PET by 4.28% after five weeks of cultivation [76]. *Nocardioides marinus* isolated from Pacific Ocean deep-sea sediments could reduce the weight of PET by 1.2–1.3% in 30 days [69]. *Gordonia sihwensis*, *Gordonia mangrovi*, and *Gordonia bronchialis* isolated from mangroves showed PS degradability with 4.69–7.73% degradation rates in one month [73]. *Srreptomyces gougerotti*, *Micromonospora matsumotoense*, and *Nocardiopsis prasina* isolated from the ocean could degrade LDPE, PS, and polylactic acid (PLA) to varying degrees [77].

#### 3.1.5. Other Bacterial Species

Many other bacterial species of Proteobacteria, Firmicutes, and Actinobacteria can degrade plastics. *Lysinibacillus* sp. JJY0216 isolated from soil grooves degraded 9% and 4% of PE and PP, respectively, in 26 days [78]. *Achromobacter denitrificans* Eb113 laiyuandi showed weight losses of 6.5% and 22.3% for LDPE and PVC, respectively, in one month [79]. *Alcaligenes faecalis* (MK517568) degrades LLDPE by 3.5%, HDPE by 5.8%, and Polyester by 17.3% after 40 days of incubation [80]. *Meyerozyma guilliermondii* and *Serratia marcescens* isolated from the gut of wax worms showed PE weight losses of 13.9% and 3.57% within 60 days, respectively [81]. *Brevibacillus brevis* from soil could degrade nylon 6, 6 microplastics with a weight loss of approximately 22 *w*/*w*% after 35 days of incubation [82]. *Acinetobacter bacterium* isolated from larvae of *Tribolium castaneum* could degrade PS, causing 12.14% weight loss after 60 days of incubation [83]. *Marinobacter gudaonensis*, *Thalassospira xiamenensis*, and *Marinobacter sedimimum* isolated from Pacific Ocean deep-sea sediments could reduce the weight of PET by 1.2–1.3% in 30 days [69].

### 3.2. Fungi

Fungi are important in environmental plastic degradation, as they move through substrates with their filamentous network structure, exploring and growing in places that are more difficult for other microorganisms to reach [84]. Previously, biodegradation by fungi with polyesterase activities has been reported, which are solely terrestrial fungi. Several marine plastic-degrading fungi have been isolated [85]. For example, the marine red yeast *Rhodotorula mucilaginosa* isolated from plastic debris in the North Sea can efficiently degrade PE using an isotopically labelled method [86]. *Penicillum* spp. isolated from the Red Sea Coast could attach to LDPE films and grow in abundance [87]. The marine fungus *Alternaria alternata* FB1, which was isolated from plastic debris, can degrade PE by forming holes across the film [84]. *Aspergillus* sp. isolated from the Bay of Bengal could cause 22% weight loss of PE in six weeks [88]. *Zalerion maritimum* isolated from Portuguese coastal waters was particularly active in degrading PE microplastics, with a mass change of 56.7% in 14 days [89]. The marine fungus *Cladosporium halotolerans* 6UPA1 isolated from deep-sea sediments can colonise PU foam and degrade plastic by secreting extracellular materials [90]. To our knowledge, this is the only study on the deep-sea fungal degradation of PU.

Some terrestrial *Aspergillus*, such as *Aspergillus nomius* RH06 [91] and *Aspergillus clavantus* JASK1 [92], can degrade LDPE plastics, as evaluated by weight loss and variation in morphology, such as cracks and fissures. Other fungal species of terrestrial origin also have significant plastic degradability, including *Trichoderma* sp. [91,93], *Monascus* sp. [94], *Clitocybe* sp. [95], *Penicillum* spp. [96], and *Phanerochaete* sp. [97]. Among them, only *Penicillum* spp. and *Aspergillus* sp. of marine origin have been isolated [98], while *Trichoderma* sp., *Monascus* sp., *Clitocybe* sp., and white-rot fungi of marine origins have yet to be isolated.

### 3.3. Algae

To date, only a few algae cases have been reported on plastic degradation. Algae adsorbed on the surface produces ligninolytic and exopolysaccharide enzymes to degrade plastic [99]. The microalga *Uronema africanum* isolated from waste plastic bags in a freshwater lake was found to degrade LDPE sheets within 30 days through observations of corrosion, abrasion, groove, and ridge configurations [100]. During the investigation, cyanobacteria appeared on plastic biofilms, showing its potential to degrade plastic. Fewer cyanobacteria have been isolated to degrade plastics. For example, *Phormidium lucidum* and *Oscillatoria subbrevis* isolated from plastic debris in domestic wastewater were found to be capable of degrading LDPE, as they colonised PE and utilised carbon without any pro-oxidant additives or pre-treatment [101]. PET and PP can be degraded after 112 days of interaction with freshwater *Spirulina* isolates, as confirmed by FTIR-ATR, SEM-EDX, and tensile strength [102]. Plastic degradation by cyanobacteria isolated from marine sources has not been reported. PETase^R280A^-FLAG, produced by a marine diatom, showed the ability to degrade PET. This is the only known degradation of plastics by marine-sourced algae and provides a foundation for later use of microalgae to solve the plastic problem [103].

## 4. Plastic Biodegradation Mechanisms

### 4.1. Biofilm Formation

Plastic debris foster a biofilm on its surface once it enters the water due to the large number of microbial communities in the aquatic environment [104]. Biofilm formation is a dynamic process that usually involves microbial adhesion, secretion of extracellular polymeric substances, and microbial proliferation [105,106]. Microbial diversity in plastic biofilms is strongly influenced by environmental factors [107]. The six most common families on PS and PU plastic surfaces in the Northeast Atlantic are *Chitinophagaceae*, *Xanthobacteraceae*, *Hyphomicrobiaceae*, *Pseudoalteromonadaceae*, *Opitutaceae*, and *Burkholderiaceae* [108]. *Flavobacteriaceae* and *Rhodobacteraceae* were commonly found on biofilms of PP, PE, PET, and PVC in the Fal Estuary [109]. In the Caribbean Sea at a depth of 1 m, scientists placed various plastics (PS, PP, PET, PE, PVC) from consumer products in seawater for six weeks, and the dominant community on the surface biofilms were *Pirellulaceae*, *Flavobacteriaceae*, *Rhodobacteraceae*, and *Saprospiraceae* [110]. *Flavobacteriaceae*, *Planctomucetaceae*, and *Colwelliaceae* were found on the surface of PVC plastic biofilms in the Mediterranean Sea [111]. Community structure can vary in different locations of the same region. Different features in the biofilm diversity of PP, PS, and PE were observed at three sites in the intertidal zone of the Yangtze estuary. *Erythrobacteraceae*, *Sphingomonadaceae*, *Comamonadaceae*, *norank_c_Cyanobacteria*, and *Blastocatellaceae_Subgroup_4_* are predominant on Chongming Island [112]. *Rhodobacteraceae*, *Erythrobacteraceae*, *Moraxellaceae*, *FamilyI_o_SubsectionIII*, and *Planococcaceae* were observed in the Lvsi port, and the most frequent families included *norank_c_Cyanobacteria*, *Saprospiraceae*, *Pseudoalteromonadaceae*, *Flavobacteriaceae*, and *Erythrobacteraceae* in Xiangshan Bay [112]. The bacterial diversity in plastic biofilms of different marine environments is summarised in Table 2.

### 4.2. Enzymes

Enzymatic biocatalysis is crucial in the biodegradation of plastics and offers a green alternative for plastic recycling [125]. Enzymes are involved in the biodegradation of plastics by first being adsorbed on the film surface with the help of the surface binding domain, and then breaking the chemical bond. Among them, PET-degrading enzymes have been most intensively investigated. In 2005, it was first reported that hydrolases isolated from the actinomycete *Thermobifida fusca* could reduce weight by 50% after three weeks of incubation [126]. Since then, a series of PET-degrading enzymes have been characterised, mostly including carboxylic ester hydrolases (cutinases, carboxylesterase, and lipases), PETase, and MHETase [125,127]. Among them, PETase and cutinases have received extensive attention. The PET hydrolase named PETase was discovered from *Ideonella sakaiensis* in 2016 and has optimal degradation activity at 40 °C [41]. To improve the depolymerisation ability, several methods (chemical modification, PET pre-treatment, protein engineering, etc.) have been used to improve the stability and activity of PET hydrolases. The best variant, DepoPETase, was screened by directed evolution that can produce 1407-fold products towards amorphous PET film at 50 °C and showed a 23.3 °C higher Tm value than the original PETase [128]. The activity and thermostability of PETase from *I. sakaiensis* were enhanced by modulating post-translation glycan modification, which can completely depolymerise untreated PET plastic within 2–3 days at 50 °C [129].

Numerous cutinases have been identified that can also degrade PET. In 2012, PET-degrading cutinase (LCC) was isolated from leaf-branch compost with the highest activity at pH 8.5 and 50 °C [130]. Subsequently, the more thermally stable enzyme can degrade at least 90% of the PET within 10 h by replacing the divalent metal binding site with a disulphide bridge [131]. Another study showed that two fusion proteins constructed by a carbohydrate-binding module and leaf-branch compost cutinase could increase the degradation efficiency of PET films by 3.7% and 24.2%, respectively [132].

Esterase is the main class of enzymes for PU degradation. In 1998, the PUR esterase isolated from *Comamonas acidovorans* TB-35 could adsorb to the PUR surface to hydrolyse its ester bonds. The optimum pH of this enzyme was 6.5, and the optimum temperature was 45 °C [133]. Subsequently, the polyurethanase isolated from *Pseudomonas* spp. (*Pseudomonas fluorescens*, *Pseudomonas chlororaphis*, and *Pseudomonas* sp. AKS31) was shown to be involved in PU degradation [134,135,136]. PE, PS, PP, and PVC are extremely difficult to degrade because of the C-C backbone compared to PET and PU, which contain hydrolysable bonds [137].

To date, studies on the characterisation of specific enzymes for PE, PS, PP, and PVC are relatively scarce. Peroxidase, laccase, manganese superoxide dismutase, and alkane hydroxylase were reported to be involved in the oxidation and depolymerisation of PE [138,139]. For example, laccase from the Antarctic sea *Psychrobacter* sp. NJ228 can reduce the mass by 13.2% after 24 h incubation at 30 °C [140]. Similarly, oxidoreductases, laccase, and lipase are involved in the PS degradation pathway. In 1997, the degradation of polystyrene by hydroquinone peroxidase isolated from *Azotobacter beijerinckii* HM121 was first reported [141]. Alkane hydroxylases and monooxygenases were found to break C-C bonds; ring-hydroxylating dioxygenases may break PS side chains to produce aromatic ring compounds, and they are potential enzymes for PS depolymerisation [142]. The key enzymes responsible for plastic biodegradation and the corresponding metabolic products are summarised in Table 3.

### 4.3. Biodegradation Mechanisms of Specific Plastics

#### 4.3.1. PS Biodegradation Mechanism

Polystyrene (PS), which is an aromatic polymer composed of styrene monomers, was first extracted from natural resins by German Eduard Simon in 1839 and has been commercially produced since 1930 [152,153]. The structure of PS can thus be written as [CH_2_CH (C_6_H_5_)] n, and it is a rigid, amorphous thermoplastic polymer [154]. To improve specific properties, PS polymers can be chemically modified with other polymers, such as styrene acrylonitrile (SAN) and acrylonitrile butadiene styrene (ABS). PS is widely used to produce CDs, toys, food storage, and various packing products due to its high translucency, durability, low cost, etc. According to the latest report, the global production of polystyrene (PS) reached 20.82 million tons in 2022 (https://www.plasticseurope.org/en, accessed on 28 December 2023), but the unique structure of PS, with aromatic rings in its polymer chain, causes its biodegradation to be very difficult [155]. Therefore, PS biodegradation has become a critical global environmental issue.

**Figure 3:**
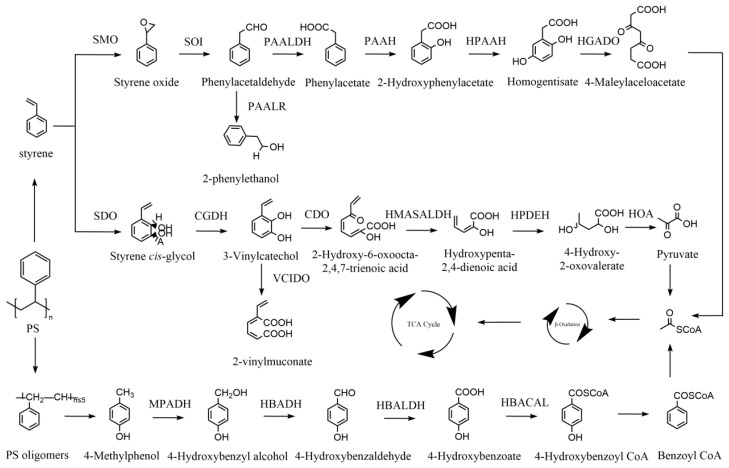
The metabolic pathways of polystyrene (PS) plastics.

Although many studies have demonstrated the biodegradation of PS by various microorganisms, the critical enzymes involved in the initial depolymerisation have not been reported [156,157]. Microbial degradation of styrene monomers has been well studied (Figure 3), including two potential metabolic pathways, the styrene cis-glycol–3-vinylcatechol and styrene oxide–phenylacetaldehyde pathways [158,159]. The first is that styrene is hydroxylated by styrene dioxygenase (SDO) on the aromatic ring to generate styrene cis-glycol, which can be further converted to acetyl-CoA by cis-glycol dehydrogenase (CGDH), catechol 2,3-dioxygenase (CDO), 2-hydroxymuconic acid semialdehyde hydrolase (HMASALDH), 2-hydroxypenta-2,4-dienoate hydratase (HPDEH), 4-hydroxy-2-oxovalerate aldolase (HOA), and the pyruvate dehydrogenase complex (PDHC), which then enter the TCA cycle. The second one was converted into styrene oxide by styrene monooxygenase (SMO). Styrene oxide is then degraded by styrene oxide isomerase (SOI) to form phenylacetaldehyde. Finally, the product was degraded into 4-maleylaceloacetate by henylacetaldehyde dehydrogenase (PAALDH), phenylacetate hydroxylase (PAAH), 2-hydroxyphenylacetate hydroxylase (HPAAH), and homogentisate 1,2-dioxygenase (HGADO), which was further converted to acetyl-CoA through the β-oxidation pathway. The styrene oxide-phenylacetaldehyde metabolic pathway has been validated for the degradation of polystyrene by larvae of the greater wax month, and another metabolic pathway has also been proposed [3]. Another potential metabolic pathway was that polystyrene produces PS oligomers, which then produce 4-methylphenol. 4-Methylphenol can be converted to benzoyl CoA by 4-methylphenol methylhydroxylase (MPMH), 4-hydroxybenzyl alcohol dehydrogenase (HBADH), 4-hydroxybenzaldehyde dehydrogenase (HBALDH), 4-hydroxybenzoic acid-CoA ligase (HBACAL), and 4-hydroxybenzoyl-CoA reductase (HBCAD). Through the β-oxidation pathway, benzoyl CoA is further converted to acetyl-CoA and then enters the TCA cycle for complete mineralisation. Several studies have demonstrated that PS degradation produces the monomer styrene [160,161]. Moreover, a novel approach to the recycling of polystyrene was reported in which styrene is converted to polyhydroxyalkanoate (PHA) by *Pseudomonas putida* CA-3 [162], which offers the possibility to solve the pollution of PS in the environment.

The enzymes responsible for each step are indicated as follows: styrene dioxygenase (SDO), cis-glycol dehydrogenase (CGDH), catechol 2,3-dioxygenase (CDO), 2-hydroxymuconic acid semialdehyde hydrolase (HMASALDH), 2-hydroxypenta-2,4-dienoate hydratase (HPDEH), 4-hydroxy-2-oxovalerate aldolase (HOA) and pyruvate dehydrogenase complex (PDHC), 2,3-vinylcatechol intradiol dioxygenase (VCIDO), styrene monooxygenase (SMO), styrene oxide isomerase (SOI), henylacetaldehyde dehydrogenase (PAALDH), phenylacetate hydroxylase (PAAH), 2-hydroxyphenylacetate hydroxylase (HPAAH), homogentisate 1,2-dioxygenase (HGADO), 4-methylphenol methylhydroxylase (MPMH), 4-hydroxybenzyl alcohol dehydrogenase (HBADH), 4-hydroxybenzaldehyde dehydrogenase (HBALDH), 4-hydroxybenzoic acid-CoA ligase (HBACAL), 4-hydroxybenzoyl-CoA reductase (HBCAD). Adapted from [3,158,159].

#### 4.3.2. PET Biodegradation

PET is a condensation of terephthalic acid (TPA) and ethylene glycol (EG) [163]. The first patent for preparing PET was published in 1946, and it achieved worldwide industrial production in 1953. With the development of material science, PET is one of the most common synthetic polymers in every aspect of our life due to it being colourless (transparent or translucent), lightweight, thermoplastic, robust, semirigid to rigid, and low toxicity [84]. The fibre and packaging industries, such as plastic bags, bottles, and films, are the industries with the largest PET in-use stocks [164,165]. However, the treatment of waste PET will be a challenge in the future due to consumption and disposal.

**Figure 4:**
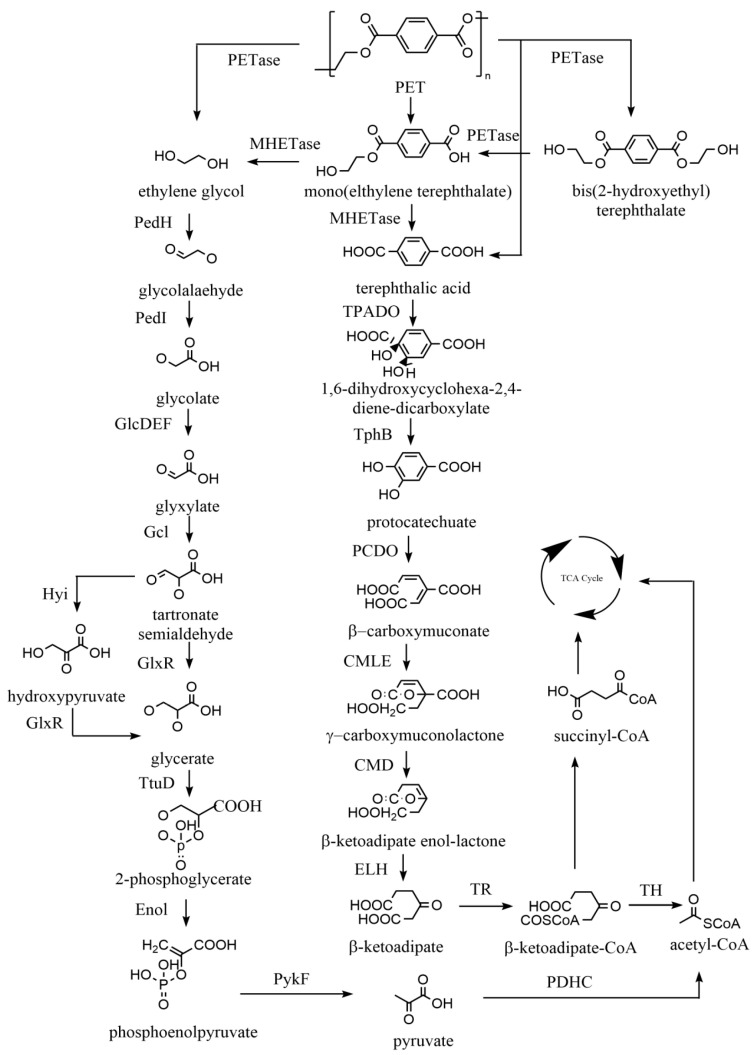
The metabolic pathways of polyethylene terephthalate (PET) plastics.

The depolymerisation of PET begins with the cleavage of ester bonds by PETase to produce ethylene glycol (EG), MHET, and BHET. As shown in Figure 4, BHET is converted to MHET by PETase, which is further converted to terephthalic acid (TPA) and EG [166]. TPA is eventually degraded into succinyl-CoA and acetyl-CoA by TPA-1,2-dioxygenase (TPADO), 1,2-dihydroxy-3,5-cyclohexadiene-1,4-dicarboxylate dehydrogenase (TphB), protocatechuate 3,4-dioxygenase (PCDO), β-carboxy-cis, cis-muconate lactonizing enzyme (CMLE), β-carboxymuconolactone decarboxylase (CMD), enollactone hydrolase (ELH), β-ketoadipate:succinyl-CoA transferase (TR), and β-ketoadipyl-CoA thiolase (TH). Similarly, EG can be further converted to acetyl-CoA by quinoprotein alcohol dehydrogenase (PedH), aldehyde dehydrogenase family protein (PedI), glycolate oxidase (GlcDEF), glyoxylate carboligase (Gcl), hydroxypyruvate isomerase (Hyi), tartronate semialdehyde reductase (GlxR), glycerate kinase (TtuD), enolase (Enol), pyruvate kinase (PykF), and pyruvate dehydrogenase complex (PDHC). The products of metabolism, succinyl-CoA and acetyl-CoA, enter the TCA cycle to provide energy for growth. In addition, the monomers generated from PET can be upgraded to higher-value chemicals and materials. For example, *Pseudomonas putida* GO16 could utilise terephthalic acid (TPA) to produce biodegradable polyhydroxyalkanoate (PHA) and hydroxyalkanoyloxy-alkanoate (HAA) [167,168]. PET hydrolysate can be used by *Rhodococcus josii* for growth and conversion to lycopene [169].

The enzymes responsible for each step are indicated as follows: TPA-1,2-dioxygenase (TPADO) 1,2-dihydroxy-3,5-cyclohexadiene-1,4-dicarboxylate dehydrogenase (TphB), protocatechuate 3,4-dioxygenase (PCDO), β-carboxy-cis, cis-muconate lactonizing enzyme (CMLE), β-carboxymuconolactone decarboxylase (CMD), enollactone hydrolase (ELH), β-ketoadipate:succinyl-CoA transferase (TR), β-ketoadipyl-CoA thiolase (TH), quinoprotein alcohol dehydrogenase (PedH), aldehyde dehydrogenase family protein (PedI), glycolate oxidase (GlcDEF), glyoxylate carboligase (Gcl),hydroxypyruvate isomerase (Hyi), tartronate semialdehyde reductase (GlxR), glycerate kinase (TtuD), enolase (Enol), pyruvate kinase (PykF), pyruvate dehydrogenase complex (PDHC). Adapted from [159,170].

#### 4.3.3. Polythene (PE) Biodegradation Mechanism

Polyethylene (PE), expressed as (C_2_H_4_) n, is a linear hydrocarbon polymer that is hydrophobic in nature [70]. It is also considered one of the most recalcitrant carbon-based synthetic materials [35]. Several properties of PE, such as high molecular weight, ductility, corrosion resistance, and durability, have caused it to be the preferred material worldwide. PE can be divided into high-density polyethylene (HDPE) and low-density polyethylene (LDPE) according to the polymerisation method, molecular weight, and chain structure. High-density polyethylene (HDPE), of which 1 kg can be produced by using 1.75 kg petroleum, has a high degree of crystallinity and has been produced since 1939 [171,172], and the polymer chain may be 500,000–1,000,000 carbon units long with little or no branching [173]. HDPE is widely used in toys, packaging, and medical supplies due to its advantages. Compared with HDPE, LDPE has a high degree of branching and low crystallinity (55–65%) and is used for films, packing, wrapping frozen food, and textile products.

**Figure 5:**
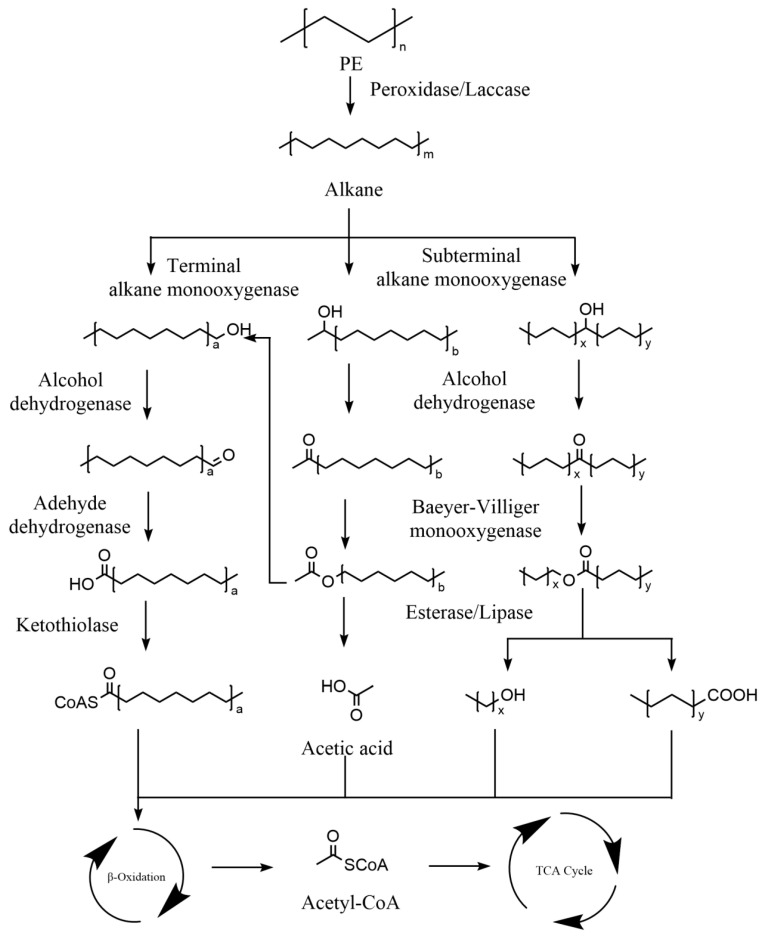
The metabolic pathways of polythene (PE) plastics. a, b, x, y represent different number of carbon atoms. Adapted from [70,84,174].

There are two major stages regarding PE biodegradation. The first stage is depolymerisation. Some enzymes, including laccase [140] and peroxidase [84], are pivotal for PE degradation. They are mainly involved in depolymerising the long carbon chains of polyethylene into oligomers, dimers, and sometimes monomer mixtures [2,174], which can re-participate in the chemical cycle of the natural environment or oligomers with 10–50 carbon atoms that are available for transportation into microorganism cells and can be further assimilated in metabolic pathways [137]. Hydroxylation is the first step for alkane assimilation [70]. As shown in Figure 5, the alkanes produced by depolymerisation could be hydroxylated by terminal oxidation monooxygenase and subterminal oxidation monooxygenase. The terminal hydroxylated alkanes eventually form fatty acids by alcohol dehydrogenase and aldehyde dehydrogenase. Furthermore, the subterminal hydroxylated alkanes are converted to esters by a Baeyer–Villiger monooxygenase and further degraded by esterase or lipase to produce fatty acids. All fatty acids produced by both pathways enter ß-oxidation to produce acetyl-CoA and finally enter the TCA cycle to complete the mineralisation process.

#### 4.3.4. Polyvinyl Chloride (PVC) Biodegradation Mechanism

PVC is a white, amorphous vinyl polymer composed of repeating ethynyls with the chemical formula C_2_H_3_Cl. It is also an inert and rigid polymer that was discovered in 1872 by the German chemist Eugen Baumann, but the first patent was received by Klatte in 1913. Due to low material costs and cost-effectiveness, it has been widely used for a variety of applications, such as food packaging, construction, water pipes, and electrical cable insulation [175]. China produces and consumes the largest amount of PVC material worldwide. It is estimated that the cumulative PVC waste in China will be 508.6 Mt by the end of 2050 [176].

PVC may lead to various environmental pollutants different from other plastics, as it is a kind of chlorine-containing plastic. Various additives, such as phthalates, carboxylates, epoxides, and polyesters, are added for durability and corrosion resistance [177]. Most of these additives are toxic and can be emitted to the surroundings, which has raised particular concerns.

**Figure 6:**
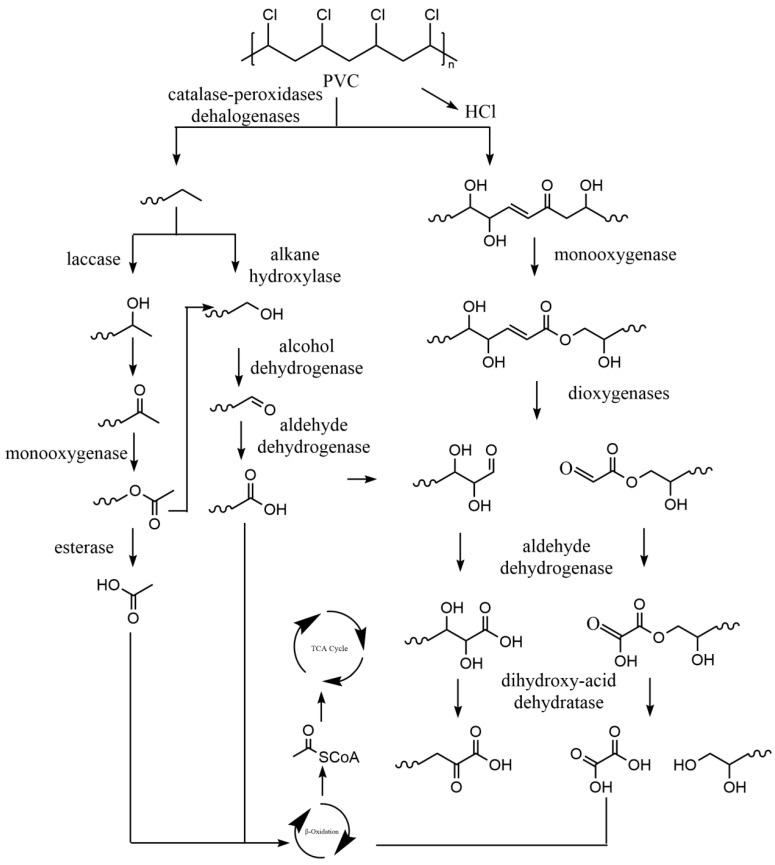
The metabolic pathways of polyvinyl chloride (PVC) plastics. Adapted from [180,182].

Microbial degradation of PVC has been observed. For example, the bacterial consortia enriched from the *Tenebrio molitor* larvae gut can degrade PVC polymers [178,179]. The strain *Klebsiella* sp. EMBL-1 isolated from the gut of insects can depolymerise PVC for further use as the sole energy source [180]. *Vibrio*, *Altermonas*, and *Cobetia* can cause changes in PVC morphology and chemical structure [181]. However, the mechanism of PVC biodegradation is poorly understood because it has no hydrolysable ester bond, which causes its degradation to be more difficult. As shown in Figure 6. Catalase-peroxidase promotes the degradation of depolymerised PVC into lower molecular weight polymers [180]. Lower molecular weight polymers are transformed into fatty acids by monooxygenase, esterase, dioxygenase, aldehyde dehydrogenase, and dihydroxy-acid dehydratase. Fatty acids are converted to acetyl-CoA by the β-oxidation pathway and then enter the TCA cycle to provide energy for growth. PVC has also been found to produce HCl as well as other chlorinated compounds in the presence of dehalogenases [40,179].

#### 4.3.5. Polyurethane (PU) Biodegradation Mechanism

Polyurethanes are synthetic polymers that carry urethane (or carbamate) bonds (-NH-COO-) in their main chains [183]. The bond is formed by step-growth polymerisation through the condensation of polyisocyanate and short- or long-chain polyols [184,185]. The most commonly used isocyanate is 4,4′-methylene diphenyl isocyanate (MDI), followed by aliphatic isocyanates [186]. The global PU market is expected to grow at a compound annual growth rate of 3.8% from 2021 to 2028 [187]. Polyurethanes are highly resistant to high temperature and hydrophobic, resulting in a long service life [188]. The hardness and elasticity of polyurethanes can be tailored by controlling the ratio of each, causing them to be widely used [189]. Flexible PU is a very good material widely used in packing and filtering [190]. Because of its nontoxicity and ability to support cell adhesion and proliferation of human cells, PU has been widely applied in the field of biomedical materials such as tissue engineering, breast surgery, and drug delivery carriers [191]. Rigid PU retains many of its merits, such as heat insulation, corrosion resistance, and sound insulation, and it has many application areas, including construction, automobiles, and aviation.

**Figure 7:**
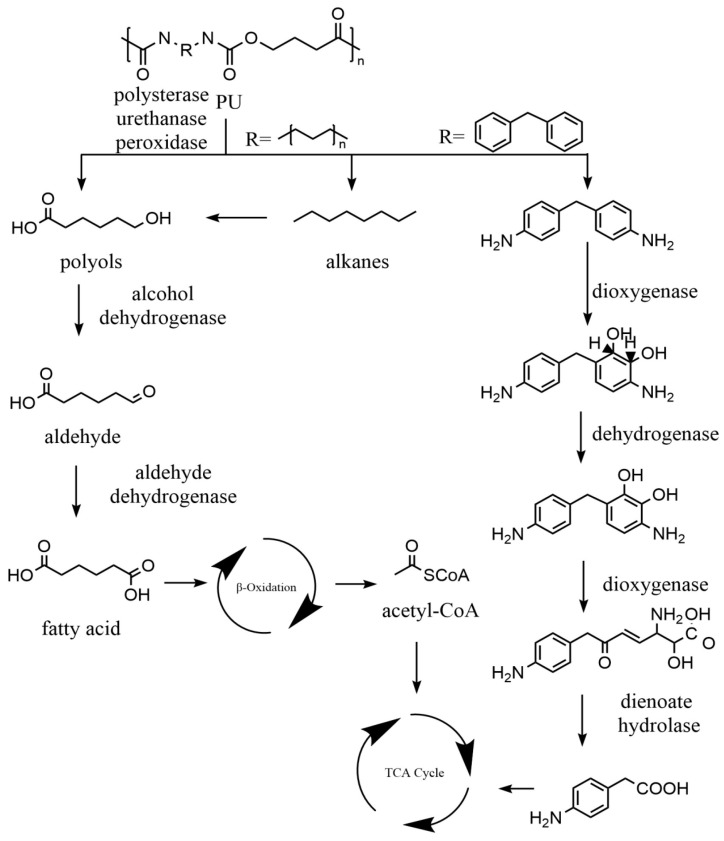
The metabolic pathways of polyurethane (PU) plastics. Adapted from [90,182].

Currently, bacteria and fungi can biodegrade PU mainly through the hydrolysis of ester bonds by polyesterases [192]. *Serratia* sp. HY-72 isolated from the gut of the Asian mantis can degrade PU (polyester- and polyether-PU), causing changes in the PU surface morphology and structure [193]. *B. subtilis* MZA-75 and *P. aeruginosa* MZA-85 isolated from soil in the form of a consortium produce large amounts of esterase to degrade PU [192]. However, it is generally believed that bacteria are less effective in degradation than fungi in PU hydrolysis. Fungal strains *Cladosporium* sp., *Rhizopus oryzae*, and *Alternaria alternate* were verified to degrade PU [90,194,195]. The structural diversity of PU leads to a variety of metabolites and metabolic pathways. As shown in Figure 7, polyesterases, urethanase, and peroxidases attack the ester and urea bonds of PU for depolymerisation, producing alkanes, 4,4′-methylenedianiline (MDA) and polyols, which are further degraded to acid by hydrolases, dioxygenases, and dienoate hydrolase. The intermediates of PU eventually enter the fatty acid metabolic pathway and the TCA cycle to achieve complete metabolism.

#### 4.3.6. Polypropylene (PP) Biodegradation Mechanism

Polypropylene (PP) was first condensed by propylene in 1954, and its production has gradually increased since the 1980s. At present, it is the second largest thermoplastic worldwide [196]. PP, expressed as (C_3_H_6_) n, is white waxy, transparent, and light and is a linear hydrocarbon resin, rigid crystalline thermoplastic, highly stable, and takes a long time to degrade. Owing to its advantageous characteristics, including low cost and density, it is one of the most widely used polymers present in everyday objects such as medical instruments, automobiles, biomedical supplies, energy supplies, machine parts, and electronic packaging [197]. In recent years, the rapid development of packaging, electronics, automobiles and other industries has greatly promoted the development of China’s industry. Moreover, with the aim of inhibiting the rapid spread of COVID-19, personal protection equipment, such as ventilators, hair, and shoe nets, is used by healthcare professionals, mainly made up of PP, at approximately 72% [198]. Therefore, the degradation of polypropylene is a difficult task.

According to previous studies, the degradation mechanism of PP is similar to that of PE, i.e., the polymer is converted to oligomers by increased oxygenated functional groups, chain scission, and subsequent methyl ketones [4]. However, there are no enzymes obtained that can degrade PP efficiently, and little has been reported about the mechanism of PP degradation.

In the marine environment, the degradation efficiency of plastics can differ due to microbial species and numbers, temperature, dissolved oxygen, etc. For example, *Pseudomonas* spp. isolated from seawater in Toyama Bay (320 m depth) can degrade PCL at 4 °C [199]. *Brevibacillus thermoruber* isolated from Bulgarian hot spring degrades PCL at 55 °C, with a weight loss of 19.8% over four weeks [200]. Four marine communities can degrade PVC in anaerobic conditions with 5.77–11.67% weight loss [201]. Microorganisms can degrade different types of plastics in different environments. However, to our knowledge, the biodegradation mechanisms of PET, PE, PP, PS, PVC, and PU are basically identical and are initiated by oxidation.

## 5. Summary and Prospects

Plastic products provide comfort and convenience in daily life, but marine pollution caused by plastics has adverse environmental, social, and economic impacts. Biodegradation is one of the environmentally friendly solutions for plastic pollution, but the process is very slow. And the special ecological environment of the oceans (high salt, anaerobic, high pressure, etc.) is beneficial for finding new microbial resources to increase the plastic biodegradation rate. Pseudomonadota (Proteobacteria), Bacteroidota (Bacteroidetes), Bacillota (Firmicutes), and Cyanobacteria are widely found on marine plastic biofilms. To date, diverse plastic-degrading bacteria have been isolated from marine environments such as offshore and deep oceanic waters. In contrast, only a few marine fungi and algae have been isolated as plastic degraders. We also summarised the biodegradation mechanisms of different types of plastics, especially the associated enzymes such as cutinases, esterases, and laccases, and highlighted the necessity of exploring novel enzymes from both marine microbial resources and biotechnology to promote the process. Considering the accumulation of plastic in various marine environments and the challenges (such as low degradation efficiency, limited understanding of degradation mechanisms, limited mineralisation capacity, and environmental factors, etc.) in understanding the environmental fates in the vast ocean ecosystem, the plastic biodegradation and fragmentation mediated by marine organisms need more investigation.

(1) The microplastics and nanoplastics resulting from the biodegradation of plastics should be considered. Although plastics can be degraded by microorganisms, they remain in a transitional state in the form of microplastics and nanoplastics for long periods before complete mineralisation. Therefore, the contribution of marine microorganisms to microplastic production during plastic colonisation and biodegradation needs to be evaluated.

(2) The effects of environmental factors (mechanical, temperature, pH, etc.) on microbial degradation should be considered to mimic the in situ degradation process, such as incorporated studies of sunlit water surfaces, cold, dark, deep-sea water columns, and sediments.

(3) Hydrocarbon-biodegrading bacteria are widely distributed in the ocean, and their potential for plastic degradation should be assessed. A relatively high percentage of marine plastic-degrading bacteria are associated with hydrocarbon degradation [114,118,202], and degradation mechanisms may be widely shared among pathways of the two kinds of molecules.

(4) More efforts are needed to explore extreme marine environments for novel microbial resources to address plastic pollution. The pelagic deep sea may provide a unique habitat for plastic-eating microbes, with immense novel diversity to be revealed, and offer opportunities to obtain unique enzymes for plastic waste recycling use. Deep-sea in situ enrichment can be carried out to excavate bacteria with plastic-degrading potential.

## Figures and Tables

**Table 1:** The common plastic waste recycling technologies.

Method	Advantages	Disadvantages	Reference
Chemical recycling	Sustainability Converted into chemical raw materials	Costly active catalysts Energy-consuming Produce toxic products	[35,38]
Incineration	Low cost Large-scale disposal	Release toxic compounds Massive CO_2_ emissions	[8,35]
Landfilling	Simple Low cost	Unsustainability Polluted soil and groundwater	[36]
Mechanical reprocessing	Cost-effective Commonly used Flexible feedstock supply	Pre-sorting Damage mechanical performances	[37]

**Table 2:** Microbial diversity in plastic biofilms listed according to marine sampling sites and plastic types.

Plastic Types	Core Communities	Sampling Sites	References
PP	*Flavobacteriaceae*, *Rhodobacteraceae*, *Rhodothermaceae*, *Erythrobacteraceae*	Four marine aquaculture sites along the southeast coast of China	[54]
	*Burkholderiales*, *Enterobacterales*, *Flavobacteriaceae*, *Pseudomonadales*, *Rhodobacteraceae*	Fal Estuary	[109]
	*Pirellulaceae*, *Flavobacteriaceae*, *Rhodobacteraceae*, *Saprospiraceae*	Caribbean Sea	[110]
	*Erythrobacter*, *Matibacter*, *Pseudoalteromonas*	Mondego estuary	[113]
	Bacteroidia, Gammaproteobacteria, Alphaproteobacteria	Mediterranean Sea	[114]
	*Psychrobacter*, *Pseudomonas*, *Flavobacterium*, *Winogradskyella*	Coastal area of Busan City	[115]
	*Flavobacteriales*, *Rhodobacterales*, *Chitinophagales*, *Rickettsiales*, *Cytophagales*, *Oceanospirillales*, *Alteromonadales*	Island of Elba in the Mediterranean Sea	[116]
	*Bacteroidales*, *Verrucomicrobiales*, *Clostridiales*, *Rhodobacterales*, *Xanthomonadales*, *Desulfovibrionales*	Freshwater Lake of Hungary	[117]
PET	Pseudomonadota (Proteobacteria), Bacteroidota (Bacteroidetes), Bacillota (Firmicutes), Cyanobacteria	Western South Atlantic	[53]
	*Flavobacteriaceae*, *Rhodobacteraceae*, *Rhodothermaceae*, *Erythrobacteraceae*	Four marine aquaculture sites along the southeast coast of China	[54]
	*Burkholderiales*, *Enterobacterales*, *Flavobacteriaceae*, *Pseudomonadales*, *Rhodobacteraceae*	Fal Estuary	[109]
	*Pirellulaceae*, *Flavobacteriaceae*, *Rhodobacteraceae*, *Saprospiraceae*	Caribbean Sea	[110]
	Pseudomonadota (Proteobacteria), Bacteroidota (Bacteroidetes), Cyanobacteria	Northern European waters	[118]
PS	Pseudomonadota (Proteobacteria), Bacteroidota (Bacteroidetes), Bacillota (Firmicutes), Cyanobacteria	Western South Atlantic	[53]
	*Flavobacteriaceae*, *Rhodobacteraceae*, *Rhodothermaceae*, *Erythrobacteraceae*	Four marine aquaculture sites along the southeast coast of China	[54]
	*Chitinophagaceae*, *Xanthobacteraceae*, *Hyphomicrobiaceae*, *Pseudoalteromonadaceae*, *Opitutaceae*, *Burkholderiaceae*	North-East Atlantic	[108]
	*Pirellulaceae*, *Flavobacteriaceae*, *Rhodobacteraceae*, *Saprospiraceae*	Caribbean Sea	[110]
	*Erythrobacter*, *Matibacter*, *Pseudoalteromonas*	Mondego estuary	[113]
	*Pseudoalteromonas*, *Maribacter*, *Erythrobacter*	Adriatic Sea	[119]
	*Psychrobacter*, *Pseudomonas*, *Flavobacterium*, *Winogradskyella* *Flavobacteriales*, *Rhodobacterales*, *Chitinophagales*, *Rickettsiales*, *Cytophagales*, *Oceanospirillales*, *Alteromonadales*	Coastal area of Busan City Island of Elba in the Mediterranean Sea	[115] [116]
PE	Pseudomonadota (Proteobacteria), Bacteroidota (Bacteroidetes), Bacillota (Firmicutes), Cyanobacteria	Western South Atlantic	[53]
	*Flavobacteriaceae*, *Rhodobacteraceae*, *Microtrichaceae*, *Pirellulaceae*	An offshore aquaculture area in Yantai City, Shandong Province	[105]
	*Burkholderiales*, *Enterobacterales*, *Flavobacteriaceae*, *Pseudomonadales*, *Rhodobacteraceae*	Fal Estuary	[109]
	*Pirellulaceae*, *Flavobacteriaceae*, *Rhodobacteraceae*, *Saprospiraceae*	Caribbean Sea	[110]
	*Erythrobacter*, *Matibacter*, *Pseudoalteromonas*	Mondego estuary	[113]
	*Psychrobacter*, *Pseudomonas*, *Flavobacterium*, *Winogradskyella*	Coastal area of Busan City	[115]
	Water: *Acinetobacter*, *Sphingomicrobium*, *Erythrobacter*, Water-sediment: *Saccharibacteria_genera_incertae_sedis*, *Alcanivorax*, *Bacillariophyta* Sediments: *Desulfatiferula*, *Aquabacterium*, *Sulfurimonas*	Dongzhaigang Mangrove	[120]
	*Flavobacteriales*, *Rhodobacterales*, *Chitinophagales*, *Rickettsiales*, *Cytophagales*, *Oceanospirillales*, *Alteromonadales*	Island of Elba in the Mediterranean Sea	[116]
	*Pseudoalteromonas*	Adriatic Sea	[121]
PVC	*Burkholderiales*, *Enterobacterales*, *Flavobacteriaceae*, *Pseudomonadales*, *Rhodobacteraceae*	Fal Estuary	[109]
	*Pirellulaceae*, *Flavobacteriaceae*, *Rhodobacteraceae*, *Saprospiraceae*	Caribbean Sea	[110]
	*Flavobacteriaceae*, *Planctomucetaceae and Colwelliacea*	Mediterranean Sea	[111]
	*Marivita*, *Ruegeria*, *Actibacter*, *Nautella*, *Erythrobacter*	Coastal water of Yantai, Shandong Province	[122]
	*Vibrio*, *Alteromonas*, *Pseudoalteromonas*	South coast of India	[123]
PU	*Chitinophagaceae*, *Xanthobacteraceae*, *Hyphomicrobiaceae*, *Pseudoalteromonadaceae*, *Opitutaceae*, *Burkholderiaceae*	North-East Atlantic	[108]
	Pseudomonadota (Proteobacteria), Bacteroidota (Bacteroidetes), Bacillota (Firmicutes), Cyanobacteria	Western South Atlantic	[53]
	*Erythrobacter*, *Matibacter*, *Pseudoalteromonas*	Mondego estuary	[113]
BP	Pseudomonadota (Proteobacteria), Bacteroidota (Bacteroidetes), Actinomycetota (Actinobacteria), Bacillota (Firmicutes), Patescibacteria, Cyanobacteria, Verrucomicrobiota (Verrucomicrobia), Desulfobacterota	Wuyuan Bay	[124]

**Table 3:** Key enzymes in involved in biodegradation of different types of plastics.

Plastic Types	Microbial Species	Sources	Enzymes	Degradation Products	References
PHB	*Pseudomonas* sp.	Activated sludge	Depolymerase	3-hydroxybutyric acid(3-HB) monomer	[143]
	*Agrobacterium* sp. DSGZ	Sewage	Depolymerase	Hydroxybutyric acid (HB) monomer, HB-HB dimers	[144]
PET	*Stenotrophomonas maltophilia* PRS8	Soil of a landfill	Cutinase-like enzyme	Terephthalic acid (TPA), mono-(2-hydroxyethyl) terephthalate (MHET), bis-(2-hydroxyethyl) terephthalate (BHET)	[145]
	*Streptomyces scabies*	Plant	Cutinase	Terephthalic acid	[146]
	*Pseudomonas aestusnigri*	Marine	Polyester Hydrolase	Mono-(2-hydroxyethyl) terephthalate (MHET)	[55]
	*Streptomyces* sp. SM14	Marine Sponge-derived	Hydrolyzing enzyme	Terephthalic acid (TPA), mono-(2-hydroxyethyl) terephthalate (MHET), bis-(2-hydroxyethyl) terephthalate (BHET), soluble di-aromatic oligomers	[147]
PBAT	*Fusarium solani*	Phytopathogenic	Cutinase	PBAT: Terephthalic acid (TPA), 1,4-butenediol terephthalic acid (BD-TPA)	[148]
	*Bacillus pumilus*	Soils	Hydrolase	Terephthalic acid, adipic acid, 1,4-butanediol	[149]
PCL	*Fusarium solani*	Phytopathogenic	Cutinase	6-hydrox hexanoic acid (6HH)	[148]
PBS	*Fusarium solani*	Phytopathogenic	Cutinase	succinic acid (SA)	[148]
PLA	-	Environmental metagenomes	Polyesterases	Lactic acid monomers, dimmers, and longer oligomers	[150]
PVC	*Cochliobolus* sp.	Plastic dumped Soils	Laccases	Aromatic compounds, polycyclic aromatic hydrocarbons	[151]

## Data Availability

Not applicable.
